# 6-Nitro­benzimidazolium dihydrogen phosphate 6-nitro­benzimidazole solvate dihydrate

**DOI:** 10.1107/S1600536810023603

**Published:** 2010-06-23

**Authors:** Zhi-Dong Shao, Xiao Jiang, Shao-Min Lan, Wen-Jing Di, Yun-Xiao Liang

**Affiliations:** a State Key Lab. Base of Novel Functional Materials and Preparation Science, Faculty of Materials Science and Chemical Engineering, Ningbo University, Ningbo, Zhejiang, 315211, People’s Republic of China

## Abstract

In the crystal structure of the title compound, C_7_H_6_N_3_O_2_^+^·H_2_PO_4_^−^·C_7_H_5_N_3_O_2_·2H_2_O, the components are connected through O—H⋯O, N—H⋯O and O—H⋯N hydrogen-bonding inter­actions, forming a sheet-like structure parallel to (101). Adjacent sheets are further linked together by strong O—H⋯O hydrogen-bonds involving the dihydrogenphosphate groups. π–π stacking inter­actions between neighbouring aromatic constituents [centroid–centroid distance 3.653 (3) Å] help to consolidate the crystal packing.

## Related literature

For the preparation of inorganic metal phosphates, see: Benard *et al.* (1996 ▶); Jensen *et al.* (2000 ▶). For template synthesis of phosphates, see: Sameski *et al.* (1993 ▶); Lii *et al.* (1998 ▶). For phosphates with organic cations, see: Dakhlaoui *et al.* (2007 ▶).
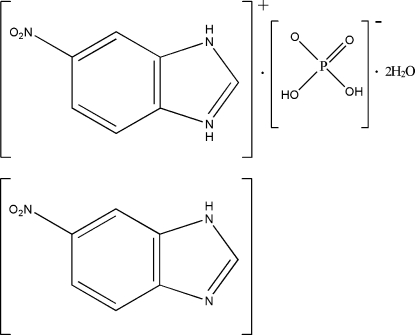


## Experimental

### 

#### Crystal data

C_7_H_6_N_3_O_2_^+^·H_2_PO_4_^−^·C_7_H_5_N_3_O_2_·2H_2_O
*M* *_r_* = 460.31
Triclinic, 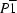

*a* = 9.4683 (19) Å
*b* = 9.990 (2) Å
*c* = 11.407 (2) Å
α = 90.73 (3)°
β = 107.10 (3)°
γ = 111.66 (3)°
*V* = 949.4 (3) Å^3^
*Z* = 2
Mo *K*α radiation
μ = 0.22 mm^−1^
*T* = 293 K
0.37 × 0.32 × 0.12 mm

#### Data collection

Rigaku R-AXIS RAPID diffractometer
Absorption correction: multi-scan (*ABSCOR*; Higashi, 1995 ▶) *T*_min_ = 0.924, *T*_max_ = 0.975
9332 measured reflections
4286 independent reflections
2827 reflections with *I* > 2σ(*I*)
*R*_int_ = 0.021

#### Refinement

*R*[*F* ^2^ > 2σ(*F* ^2^)] = 0.041
*wR*(*F* ^2^) = 0.141
*S* = 1.14
4286 reflections
280 parameters
H-atom parameters constrained
Δρ_max_ = 0.43 e Å^−3^
Δρ_min_ = −0.47 e Å^−3^

### 

Data collection: *RAPID-AUTO* (Rigaku, 1998 ▶); cell refinement: *RAPID-AUTO*; data reduction: *CrystalStructure* (Rigaku/MSC, 2002 ▶); program(s) used to solve structure: *SHELXS97* (Sheldrick, 2008 ▶); program(s) used to refine structure: *SHELXL97* (Sheldrick, 2008 ▶); molecular graphics: *ORTEPII* (Johnson, 1976 ▶) and *DIAMOND* (Brandenburg, 2008 ▶); software used to prepare material for publication: *SHELXL97*.

## Supplementary Material

Crystal structure: contains datablocks I, global. DOI: 10.1107/S1600536810023603/wm2359sup1.cif

Structure factors: contains datablocks I. DOI: 10.1107/S1600536810023603/wm2359Isup2.hkl

Additional supplementary materials:  crystallographic information; 3D view; checkCIF report

## Figures and Tables

**Table 1:** Selected bond lengths (Å)

P1—O8	1.500 (2)
P1—O7	1.504 (2)
P1—O5	1.5591 (19)
P1—O6	1.562 (2)

**Table 2:** Hydrogen-bond geometry (Å, °)

*D*—H⋯*A*	*D*—H	H⋯*A*	*D*⋯*A*	*D*—H⋯*A*
N1—H1*A*⋯O7^i^	0.86	1.74	2.600 (2)	179
N2—H2*A*⋯O9	0.86	1.92	2.752 (2)	164
O6—H6*A*⋯N4^ii^	1.03	1.66	2.665 (2)	165
O5—H5*A*⋯O8^iii^	0.91	1.62	2.531 (2)	174
N5—H5*B*⋯O7	0.86	1.91	2.773 (2)	176
O9—H9*A*⋯O4^iv^	0.91	2.43	3.161 (2)	137
O9—H9*B*⋯O10^v^	0.85	1.92	2.754 (2)	165
O10—H10*A*⋯O8	0.91	1.85	2.740 (2)	169
O10—H10*B*⋯O9^iii^	0.84	2.16	2.917 (2)	149

Symmetry codes: (i) 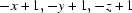; (ii) 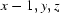; (iii) 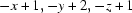; (iv) 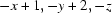; (v) 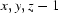.

**Fig. 1.**
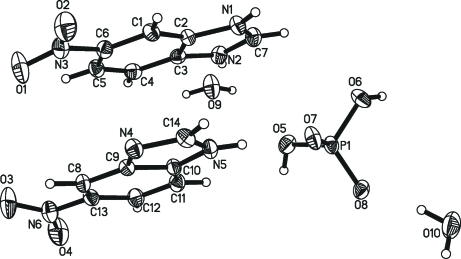
The molecular structure of the title compound, with the atomic numbering scheme. Displacement ellipsoids are drawn at the 30% probability level. H atoms are shown as small spheres of arbitrary radius.

**Fig. 2.**
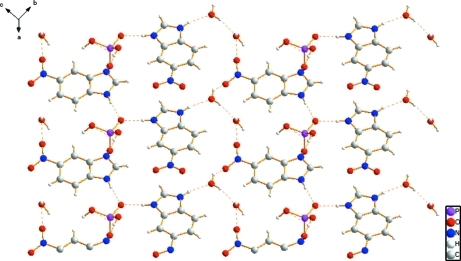
The layer structure of (C7H6N3O2)[H2PO4].(C7H5N3O2).2(H2O). Hydrogen bonds are indicated by dashed lines.

**Fig. 3.**
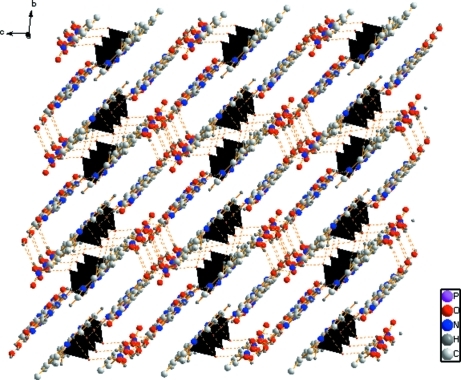
A packing diagram for the title compound, viewed along the a axis. Dashed lines indicate hydrogen bonds.

## supplementary crystallographic information

### Comment

Phosphates are of great interest because of their rich crystal chemistry and practical applications. Up to now, numerous inorganic metal phosphates have been reported (e.g. Benard *et al.*, 1996; Jensen *et al.*, 2000). Furthermore, various of these phosphates were synthesized by structure-orienting templates molecules, most ferquently amines (Sameski *et al.*, 1993; Lii *et al.*, 1998). Compared with these inorganic phosphates, the synthesis of non-metal phosphates was less well explored in the past decades (Dakhlaoui *et al.*, 2007). Herein, we describe the synthesis and crystal structure of the title compound (I), a new non-metal phosphate with formula (C_7_H_6_N_3_O_2_)[H_2_PO_4_]^.^(C_7_H_5_N_3_O_2_)^.^2(H_2_O)

As shown in Fig.1, the structure of (I) consists of one (C_7_H_6_N_3_O_2_)^+^ cation, one [H_2_PO_4_]^-^ anion, one (C_7_H_5_N_3_O_2_) solvent molecule and two H_2_O molecules, *viz*. one imidazole molecule is protonated, one imidazole molecule acts as an unprotonated solvent and a dihydrogenphosphate group is present. The O—P—O angles are in the range 105.62 (11)—115.73 (13) °. The P—O bond lengths to the terminal O atoms are 1.500 (2) and 1.504 (2) Å while the P—OH bond lengths are considerably longer with 1.5591 (19) and 1.562 (2) Å.

As is well known, hydrogen bonding interactions play an important role in the formation and stability of low-dimensional structures. In the present structure, the [(C_7_H_6_N_3_O_2_)]^+^ cations, [H_2_PO_4_]^-^ anions, (C_7_H_5_N_3_O_2_) and H_2_O molecules are linked together through hydrogen bonds: N1—H1A···O7, N5—H5B···O7; N2—H2A···O9, O6—H6A···N4, O9—H4A···O4, O9—H9B···O10, O10—H10B···O3 (Fig. 2), forming a two-dimensional sheetlike structure parallel to (101). Adjacent sheets are further linked together by strong H-bonding interactions [O5—H5A···O8, O10—H10A···O8, O10—H10B···O9]. π—π stacking interactions between neighboring 6-nitrobenzimidazole molecules with an interplanar distance of 3.653 (3) Å help to consolidate a three-dimensional supramolecular network structure (Fig. 3).

### Experimental

The title compound was obtained by the reaction of phosphoric acid, 6-nitrobenzimidazole and methanol/distilled water under room temperature. Typically, a mixture of phosphoric acid (0.2 ml), analytically pure 6-nitrobenzimidazole (0.164 g) and methanol/distilled water (10 ml/10 ml) was stirred at room temperature before it was filtered. The final filtrate was allowed to evaporate slowly at room temperature for 7 days to obtain yellow crystals.

### Refinement

All H atoms associated with C atoms and N atoms were positioned geometrically and refined as riding model, with N–H = 0.86 Å, C–H_aromatic_ type = 0.93 Å, *U*_iso_(H) = 1.2*U*_eq_(N), *U*_iso_(H) = 1.2*U*_eq_(C). Hydrogen atoms attached to O5, O6, O9 and O10 were discernible from difference Fourier maps. Their *U*_iso_(H) values were fixed at 0.05 Å^2^ and their coordinates were not refined.

### Crystal data

**Table:**

C_7_H_6_N_3_O_2_^+^·H_2_PO_4_^−^·C_7_H_5_N_3_O_2_·2H_2_O	*Z* = 2
*M**_r_* = 460.31	*F*(000) = 476
Triclinic, *P*1	*D*_x_ = 1.610 Mg m^−^^3^
Hall symbol: -P 1	Mo *K*α radiation, λ = 0.71073 Å
*a* = 9.4683 (19) Å	Cell parameters from 6404 reflections
*b* = 9.990 (2) Å	θ = 3.1–27.5°
*c* = 11.407 (2) Å	µ = 0.22 mm^−^^1^
α = 90.73 (3)°	*T* = 293 K
β = 107.10 (3)°	Platelet, yellow
γ = 111.66 (3)°	0.37 × 0.32 × 0.12 mm
*V* = 949.4 (3) Å^3^	

### Data collection

**Table:**

Rigaku R-AXIS RAPID diffractometer	4286 independent reflections
Radiation source: fine-focus sealed tube	2827 reflections with *I* > 2σ(*I*)
graphite	*R*_int_ = 0.021
ω scans	θ_max_ = 27.5°, θ_min_ = 3.1°
Absorption correction: multi-scan (*ABSCOR*; Higashi, 1995)	*h* = −12→12
*T*_min_ = 0.924, *T*_max_ = 0.975	*k* = −12→12
9332 measured reflections	*l* = −14→14

### Refinement

**Table:**

Refinement on *F*^2^	Primary atom site location: structure-invariant direct methods
Least-squares matrix: full	Secondary atom site location: difference Fourier map
*R*[*F*^2^ > 2σ(*F*^2^)] = 0.041	Hydrogen site location: inferred from neighbouring sites
*wR*(*F*^2^) = 0.141	H-atom parameters constrained
*S* = 1.14	*w* = 1/[σ^2^(*F*_o_^2^) + (0.0532*P*)^2^ + 0.7024*P*] where *P* = (*F*_o_^2^ + 2*F*_c_^2^)/3
4286 reflections	(Δ/σ)_max_ = 0.005
280 parameters	Δρ_max_ = 0.43 e Å^−^^3^
0 restraints	Δρ_min_ = −0.47 e Å^−^^3^

### Special details

**Table:**

Geometry. All e.s.d.'s (except the e.s.d. in the dihedral angle between two l.s. planes) are estimated using the full covariance matrix. The cell e.s.d.'s are taken into account individually in the estimation of e.s.d.'s in distances, angles and torsion angles; correlations between e.s.d.'s in cell parameters are only used when they are defined by crystal symmetry. An approximate (isotropic) treatment of cell e.s.d.'s is used for estimating e.s.d.'s involving l.s. planes.
Refinement. Refinement of *F*^2^ against ALL reflections. The weighted *R*-factor *wR* and goodness of fit *S* are based on *F*^2^, conventional *R*-factors *R* are based on *F*, with *F* set to zero for negative *F*^2^. The threshold expression of *F*^2^ > σ(*F*^2^) is used only for calculating *R*-factors(gt) *etc*. and is not relevant to the choice of reflections for refinement. *R*-factors based on *F*^2^ are statistically about twice as large as those based on *F*, and *R*- factors based on ALL data will be even larger.

### Fractional atomic coordinates and isotropic or equivalent isotropic displacement parameters (Å^2^)

**Table:**

	*x*	*y*	*z*	*U*_iso_*/*U*_eq_	
P1	0.46015 (8)	0.77962 (7)	0.52850 (6)	0.03448 (19)	
O1	1.0279 (3)	0.6110 (3)	0.1175 (3)	0.0759 (8)	
N1	0.4441 (3)	0.4733 (2)	0.2701 (2)	0.0392 (5)	
H1A	0.4368	0.4114	0.3221	0.047*	
C1	0.6946 (3)	0.4893 (3)	0.2258 (2)	0.0336 (5)	
H1B	0.7181	0.4226	0.2760	0.040*	
O2	0.9605 (3)	0.4445 (3)	0.2308 (2)	0.0638 (6)	
N2	0.3902 (3)	0.6274 (2)	0.1497 (2)	0.0385 (5)	
H2A	0.3417	0.6800	0.1120	0.046*	
C2	0.5632 (3)	0.5227 (3)	0.2149 (2)	0.0313 (5)	
O3	1.1886 (3)	1.0612 (3)	0.1237 (3)	0.0771 (8)	
N3	0.9352 (3)	0.5369 (3)	0.1686 (2)	0.0447 (6)	
C3	0.5284 (3)	0.6217 (3)	0.1383 (2)	0.0317 (5)	
O4	1.0632 (3)	1.1997 (3)	0.1303 (3)	0.0762 (8)	
N4	1.0431 (3)	0.7285 (2)	0.4511 (2)	0.0379 (5)	
C4	0.6251 (3)	0.6929 (3)	0.0684 (2)	0.0376 (6)	
H4B	0.6011	0.7583	0.0168	0.045*	
O5	0.4699 (3)	0.8359 (2)	0.40310 (18)	0.0489 (5)	
H5A	0.4918	0.9324	0.4014	0.050*	
N5	0.8298 (3)	0.7543 (2)	0.47608 (19)	0.0375 (5)	
H5B	0.7499	0.7403	0.5024	0.045*	
C5	0.7570 (3)	0.6616 (3)	0.0796 (2)	0.0376 (6)	
H5C	0.8259	0.7070	0.0355	0.045*	
O6	0.2931 (2)	0.6510 (2)	0.4983 (2)	0.0471 (5)	
H6A	0.1868	0.6647	0.4826	0.050*	
N6	1.0976 (3)	1.0955 (3)	0.1613 (2)	0.0492 (6)	
C6	0.7888 (3)	0.5623 (3)	0.1566 (2)	0.0347 (6)	
O7	0.5822 (2)	0.7132 (2)	0.57244 (19)	0.0456 (5)	
C7	0.3444 (3)	0.5381 (3)	0.2287 (3)	0.0425 (6)	
H7B	0.2539	0.5234	0.2518	0.051*	
O8	0.4731 (3)	0.8988 (2)	0.61749 (16)	0.0435 (5)	
C8	1.0885 (3)	0.9142 (3)	0.3032 (2)	0.0367 (6)	
H8B	1.1769	0.9045	0.2902	0.044*	
O9	0.2853 (3)	0.8166 (3)	0.0119 (2)	0.0625 (6)	
H9A	0.2025	0.8421	0.0112	0.050*	
H9B	0.3401	0.8590	−0.0339	0.050*	
C9	1.0114 (3)	0.8331 (3)	0.3813 (2)	0.0320 (5)	
O10	0.4652 (3)	0.9058 (3)	0.8556 (2)	0.0754 (8)	
H10A	0.4652	0.8914	0.7771	0.050*	
H10B	0.5564	0.9646	0.9008	0.050*	
C10	0.8767 (3)	0.8494 (3)	0.3970 (2)	0.0322 (5)	
C11	0.8168 (3)	0.9477 (3)	0.3391 (2)	0.0389 (6)	
H11A	0.7279	0.9578	0.3508	0.047*	
C12	0.8942 (3)	1.0293 (3)	0.2638 (2)	0.0401 (6)	
H12A	0.8592	1.0976	0.2244	0.048*	
C13	1.0254 (3)	1.0093 (3)	0.2469 (2)	0.0364 (6)	
C14	0.9324 (3)	0.6863 (3)	0.5052 (2)	0.0387 (6)	
H14A	0.9253	0.6161	0.5585	0.046*	

### Atomic displacement parameters (Å^2^)

**Table:**

	*U*^11^	*U*^22^	*U*^33^	*U*^12^	*U*^13^	*U*^23^
P1	0.0389 (4)	0.0324 (4)	0.0420 (4)	0.0175 (3)	0.0220 (3)	0.0148 (3)
O1	0.0584 (15)	0.097 (2)	0.104 (2)	0.0408 (15)	0.0564 (15)	0.0469 (16)
N1	0.0364 (12)	0.0418 (13)	0.0442 (13)	0.0156 (10)	0.0187 (10)	0.0156 (10)
C1	0.0338 (13)	0.0333 (13)	0.0338 (13)	0.0131 (11)	0.0109 (10)	0.0113 (10)
O2	0.0587 (14)	0.0820 (17)	0.0750 (16)	0.0482 (14)	0.0283 (12)	0.0319 (13)
N2	0.0416 (13)	0.0427 (13)	0.0403 (12)	0.0253 (11)	0.0146 (10)	0.0109 (10)
C2	0.0294 (12)	0.0323 (13)	0.0327 (13)	0.0109 (10)	0.0120 (10)	0.0063 (10)
O3	0.0843 (18)	0.103 (2)	0.0893 (18)	0.0559 (17)	0.0655 (16)	0.0499 (16)
N3	0.0375 (13)	0.0537 (15)	0.0484 (14)	0.0204 (12)	0.0182 (11)	0.0078 (11)
C3	0.0347 (13)	0.0311 (13)	0.0282 (12)	0.0131 (11)	0.0084 (10)	0.0037 (9)
O4	0.0911 (19)	0.0713 (16)	0.101 (2)	0.0464 (15)	0.0594 (16)	0.0566 (15)
N4	0.0334 (12)	0.0393 (12)	0.0444 (13)	0.0156 (10)	0.0151 (10)	0.0105 (10)
C4	0.0433 (15)	0.0337 (13)	0.0330 (13)	0.0130 (12)	0.0107 (11)	0.0087 (10)
O5	0.0798 (15)	0.0367 (10)	0.0441 (11)	0.0264 (11)	0.0341 (11)	0.0164 (8)
N5	0.0325 (12)	0.0453 (13)	0.0373 (12)	0.0135 (10)	0.0172 (9)	0.0084 (9)
C5	0.0366 (14)	0.0419 (15)	0.0325 (13)	0.0109 (12)	0.0144 (11)	0.0086 (11)
O6	0.0365 (11)	0.0321 (10)	0.0764 (14)	0.0126 (8)	0.0240 (10)	0.0171 (9)
N6	0.0469 (14)	0.0591 (16)	0.0500 (15)	0.0219 (13)	0.0252 (12)	0.0194 (12)
C6	0.0289 (13)	0.0423 (14)	0.0322 (13)	0.0149 (11)	0.0078 (10)	0.0036 (10)
O7	0.0405 (11)	0.0490 (11)	0.0649 (13)	0.0249 (9)	0.0316 (10)	0.0280 (10)
C7	0.0383 (15)	0.0491 (16)	0.0470 (16)	0.0212 (13)	0.0182 (12)	0.0106 (12)
O8	0.0659 (13)	0.0377 (10)	0.0380 (10)	0.0233 (10)	0.0281 (9)	0.0151 (8)
C8	0.0297 (13)	0.0420 (15)	0.0407 (14)	0.0125 (11)	0.0166 (11)	0.0042 (11)
O9	0.0635 (14)	0.0811 (16)	0.0725 (15)	0.0465 (13)	0.0389 (12)	0.0432 (12)
C9	0.0291 (12)	0.0320 (13)	0.0331 (13)	0.0088 (10)	0.0116 (10)	0.0031 (10)
O10	0.0840 (19)	0.100 (2)	0.0467 (13)	0.0308 (16)	0.0344 (13)	0.0136 (13)
C10	0.0298 (13)	0.0349 (13)	0.0319 (13)	0.0097 (11)	0.0133 (10)	0.0055 (10)
C11	0.0332 (14)	0.0465 (15)	0.0410 (15)	0.0189 (12)	0.0133 (11)	0.0054 (11)
C12	0.0376 (14)	0.0437 (15)	0.0426 (15)	0.0183 (12)	0.0148 (12)	0.0108 (11)
C13	0.0357 (14)	0.0355 (14)	0.0360 (14)	0.0090 (11)	0.0150 (11)	0.0075 (10)
C14	0.0367 (14)	0.0356 (14)	0.0417 (15)	0.0111 (12)	0.0132 (11)	0.0093 (11)

### Geometric parameters (Å, °)

**Table:**

P1—O8	1.500 (2)	O5—H5A	0.9100
P1—O7	1.504 (2)	N5—C14	1.348 (4)
P1—O5	1.5591 (19)	N5—C10	1.364 (3)
P1—O6	1.562 (2)	N5—H5B	0.8600
O1—N3	1.221 (3)	C5—C6	1.391 (4)
N1—C7	1.315 (4)	C5—H5C	0.9300
N1—C2	1.388 (3)	O6—H6A	1.0287
N1—H1A	0.8600	N6—C13	1.463 (3)
C1—C2	1.376 (3)	C7—H7B	0.9300
C1—C6	1.378 (3)	C8—C13	1.371 (4)
C1—H1B	0.9300	C8—C9	1.396 (3)
O2—N3	1.221 (3)	C8—H8B	0.9300
N2—C7	1.328 (3)	O9—H9A	0.9074
N2—C3	1.374 (3)	O9—H9B	0.8512
N2—H2A	0.8600	C9—C10	1.405 (3)
C2—C3	1.393 (3)	O10—H10A	0.9048
O3—N6	1.215 (3)	O10—H10B	0.8438
N3—C6	1.467 (3)	C10—C11	1.389 (4)
C3—C4	1.394 (4)	C11—C12	1.373 (4)
O4—N6	1.228 (3)	C11—H11A	0.9300
N4—C14	1.310 (3)	C12—C13	1.395 (4)
N4—C9	1.388 (3)	C12—H12A	0.9300
C4—C5	1.367 (4)	C14—H14A	0.9300
C4—H4B	0.9300		
			
O8—P1—O7	115.73 (13)	C6—C5—H5C	119.8
O8—P1—O5	109.95 (10)	P1—O6—H6A	123.8
O7—P1—O5	108.51 (11)	O3—N6—O4	122.7 (3)
O8—P1—O6	110.30 (12)	O3—N6—C13	118.9 (3)
O7—P1—O6	105.62 (11)	O4—N6—C13	118.4 (2)
O5—P1—O6	106.23 (13)	C1—C6—C5	124.3 (2)
C7—N1—C2	107.7 (2)	C1—C6—N3	117.6 (2)
C7—N1—H1A	126.2	C5—C6—N3	118.0 (2)
C2—N1—H1A	126.2	N1—C7—N2	110.9 (2)
C2—C1—C6	114.8 (2)	N1—C7—H7B	124.5
C2—C1—H1B	122.6	N2—C7—H7B	124.5
C6—C1—H1B	122.6	C13—C8—C9	115.7 (2)
C7—N2—C3	108.4 (2)	C13—C8—H8B	122.2
C7—N2—H2A	125.8	C9—C8—H8B	122.2
C3—N2—H2A	125.8	H9A—O9—H9B	116.1
C1—C2—N1	131.2 (2)	N4—C9—C8	130.5 (2)
C1—C2—C3	122.0 (2)	N4—C9—C10	109.0 (2)
N1—C2—C3	106.8 (2)	C8—C9—C10	120.4 (2)
O1—N3—O2	122.8 (3)	H10A—O10—H10B	110.3
O1—N3—C6	118.4 (2)	N5—C10—C11	132.1 (2)
O2—N3—C6	118.8 (2)	N5—C10—C9	105.6 (2)
N2—C3—C2	106.2 (2)	C11—C10—C9	122.3 (2)
N2—C3—C4	131.9 (2)	C12—C11—C10	117.3 (2)
C2—C3—C4	121.9 (2)	C12—C11—H11A	121.4
C14—N4—C9	104.8 (2)	C10—C11—H11A	121.4
C5—C4—C3	116.5 (2)	C11—C12—C13	119.7 (3)
C5—C4—H4B	121.8	C11—C12—H12A	120.1
C3—C4—H4B	121.8	C13—C12—H12A	120.1
P1—O5—H5A	115.9	C8—C13—C12	124.6 (2)
C14—N5—C10	107.1 (2)	C8—C13—N6	118.7 (2)
C14—N5—H5B	126.5	C12—C13—N6	116.7 (2)
C10—N5—H5B	126.5	N4—C14—N5	113.5 (2)
C4—C5—C6	120.4 (2)	N4—C14—H14A	123.3
C4—C5—H5C	119.8	N5—C14—H14A	123.3

### Hydrogen-bond geometry (Å, °)

**Table:**

*D*—H···*A*	*D*—H	H···*A*	*D*···*A*	*D*—H···*A*
N1—H1A···O7^i^	0.86	1.74	2.600 (2)	179
N2—H2A···O9	0.86	1.92	2.752 (2)	164
O6—H6A···N4^ii^	1.03	1.66	2.665 (2)	165
O5—H5A···O8^iii^	0.91	1.62	2.531 (2)	174
N5—H5B···O7	0.86	1.91	2.773 (2)	176
O9—H9A···O3^ii^	0.91	2.59	3.269 (2)	132
O9—H9A···O4^iv^	0.91	2.43	3.161 (2)	137
O9—H9B···O10^v^	0.85	1.92	2.754 (2)	165
O10—H10A···O8	0.91	1.85	2.740 (2)	169
O10—H10B···O9^iii^	0.84	2.16	2.917 (2)	149
O10—H10B···O3^vi^	0.84	2.61	3.106 (2)	119

Symmetry codes: (i) −*x*+1, −*y*+1, −*z*+1;  (ii) *x*−1, *y*, *z*; (iii) −*x*+1, −*y*+2, −*z*+1; (iv) −*x*+1, −*y*+2, −*z*; (v) *x*, *y*, *z*−1; (vi) −*x*+2, −*y*+2, −*z*+1.
